# New Trends in Quantitative Assessment of the Corneal Barrier Function

**DOI:** 10.3390/s140508718

**Published:** 2014-05-16

**Authors:** Anton Guimerà, Xavi Illa, Estefania Traver, Carmen Herrero, Miguel J. Maldonado, Rosa Villa

**Affiliations:** 1 Institut de Microelectrònica de Barcelona IMB-CNM (CSIC), 08193 Bellaterra, Spain; E-Mails: anton.guimera@csic.es (A.G.); rosa.villa@csic.es (R.V.); 2 CIBER-BBN, Networking Center on Bioengineering, Biomaterials and Nanomedicine, 50018 Zaragoza, Spain; 3 Laboratorios SALVAT S.A., 08950 Esplugues de Llobregat, Spain; E-Mails: etraver@salvatbiotech.com (E.T.); cherrero@salvatbiotech.com (C.H.); 4 IOBA-Eye Institute, University of Valladolid, 47011 Valladolid, Spain; E-Mail: maldonado@ioba.med.uva.es

**Keywords:** electrical impedance spectroscopy, corneal epithelium, permeability, barrier function, translayer electrical resistance, non-invasive, wound healing process

## Abstract

The cornea is a very particular tissue due to its transparency and its barrier function as it has to resist against the daily insults of the external environment. In addition, maintenance of this barrier function is of crucial importance to ensure a correct corneal homeostasis. Here, the corneal epithelial permeability has been assessed *in vivo* by means of non-invasive tetrapolar impedance measurements, taking advantage of the huge impact of the ion fluxes in the passive electrical properties of living tissues. This has been possible by using a flexible sensor based in SU-8 photoresist. In this work, a further analysis focused on the validation of the presented sensor is performed by monitoring the healing process of corneas that were previously wounded. The obtained impedance measurements have been compared with the damaged area observed in corneal fluorescein staining images. The successful results confirm the feasibility of this novel method, as it represents a more sensitive *in vivo* and non-invasive test to assess low alterations of the epithelial permeability. Then, it could be used as an excellent complement to the fluorescein staining image evaluation.

## 1. Introduction

The cornea is one of the few human tissues that is always in direct contact with the environment. This fact, together with its transparency, makes the cornea a very special tissue. In particular, the corneal epithelium, which is the outer part of the cornea, acts as a barrier against the daily insults of the external environment. Moreover, and to ensure its transparency, the cornea does not have blood vessels for its nourishment. Nutrients are supplied by diffusion through the epithelium and endothelium layer, ensuring a proper homeostasis. These properties are critically dependent [1], therefore proper and quantitative measurements of the epithelium permeability are of special clinical interest.

Nowadays, in clinical practice, the barrier function of the corneal epithelium and endothelium can be directly evaluated by measuring its permeability to fluorescein [2]. This is a non-invasive technique where the fluorescein is topically instilled and after several hours, when the dye becomes uniformly distributed through the cornea, it is measured in the cornea and anterior chamber by using a suitable fluorophotometer. Although optical fluorophotometers have evolved over the years giving pace to commercial instruments (*i.e.*, Fluorotron Master, Ocumetrics, Inc., Mountain View, CA, USA), this technique still presents substantial variability between repeated measurements. This drawback indicates that a single-drop procedure is unreliable for monitoring individual patient changes [3], which, together with the time required to perform the measurement, reduces its application to experimental purposes only.

In order to overcome the limitations of this method, our group has been focused in using the passive electrical properties of the cornea to assess the corneal barrier function [4–6]. The corneal electrical properties were firstly studied on excised lens from cow eyes by Pauly and Schwan [7]. In summary, *in vitro* analyses based on Translayer Electrical Resistance (TER) measurements have been consistently used to study the corneal permeability [8,9]. However, studies of the corneal electrical properties performed in *in vivo* conditions are quite limited as, only few reported works are available [10–12], revealing the difficulty of performing these analyses. The last approaches to perform *in vivo* measurements have adapted the existing TER measurement methods for being implemented in living animals [13–15]. However, the invasiveness of these procedures makes it impossible for being used in clinical practice.

Microfabrication technologies developed for the microelectronic industry have been extensively developed during the last thirty years. Among the wide range of applications that have taken advantage of these technologies, sensors to monitor different ocular problems have been developed. In particular, one of the major interests has been focused on monitoring the intraocular ocular pressure (IOP) to study the disease glaucoma [16,17].

**Figure 1.**
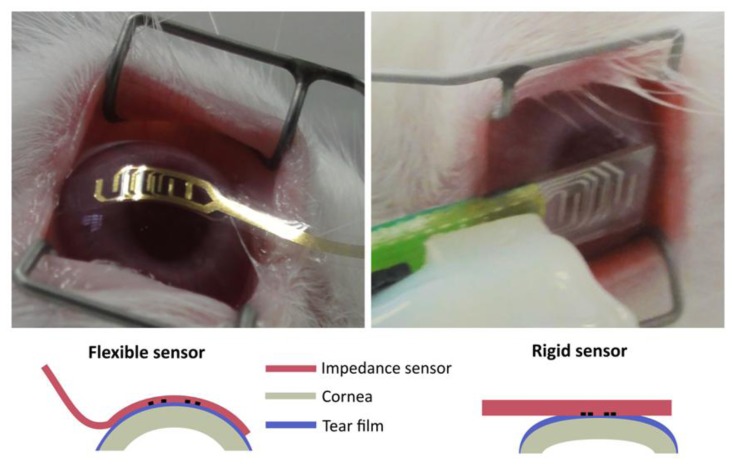
Image and sketch representation of how the impedance sensor is applied. It is interesting to note that the tear film distribution should be more homogeneous in the case of the flexible sensor.

Alternatively, there has been a lack of progress in applying these technologies for developing *in vivo* and non-invasive devices to assess the corneal barrier function. Our group studied how to solve this problem by performing tetrapolar impedance measurements with electrodes placed on the surface of the cornea [5]. This method, besides the limitations encountered on its application, was experimentally validated by using a Pyrex planar sensing device [4]. To overcome the application problems caused by the rigid nature of the sensor and taking into account the expertise that our group has in processing SU-8 photoresist, this polymer was used to fabricate a flexible sensing device. With this flexible device the application method to assess the corneal barrier function is dramatically improved, as it can be observed in Figure 1. In particular, it is possible to minimize the influence of the tear film and therefore, to discern between lower and time-dependent alterations in the corneal epithelium permeability after instillation of very low concentrated BAC solution (0.01%) [18].

In this work a further analysis focused on the validation of the flexible sensor shown in Figure 1 and presented in a previous work from the authors [18] is described. This analysis consists in monitoring the healing process of corneas that were previously wounded. For that, the obtained impedance measurements are compared with fluorescein staining images, a commonly used method to evaluate structural alterations of the corneal epithelium. The obtained results are in agreement with the fluorescein evaluation. Moreover, it has been observed that the proposed method is able to detect small alterations of the epithelial permeability that cannot be observed in the case of fluorescein staining. This is of special relevance at the final stages of the wounding process when the structural damage of the epithelium is very low. Consequently, these results confirm the feasibility of the proposed method to complement the fluorescein staining technique in the case of lower alterations of the epithelial permeability.

## 2. Experimental Section

### 2.1. Sensor Fabrication

**Figure 2.**
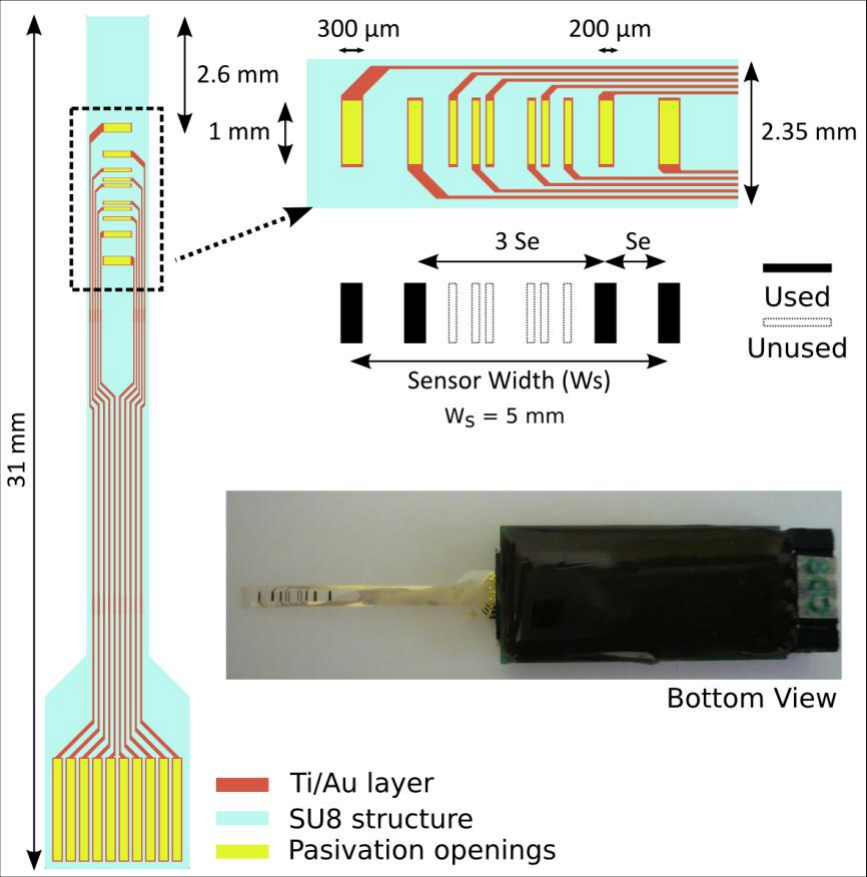
Sketch of the flexible sensor device and bottom image of the packaged sensor ready to be used with the 5 mm electrode configuration.

The electrode geometry of the SU-8 based impedance sensor that has been used in this work is shown in Figure 2. Its fabrication process was carried out in the clean room facilities at the Barcelona Microelectronics Institute (IMB-CNM). The devices were fabricated onto a silicon wafer used a carrier substrate. There, a Cr/Al (50/100 nm) bi-layer was evaporated as a sacrificial layer before depositing a first 25 μm thick SU-8 layer that was structured using a standard photolithography process. Then, gold electrodes were defined onto the SU-8 layer after evaporating a 20/200 nm Ti/Au bi-layer (where Ti acts as an adhesion layer) by using another standard photolithography process and subsequent wet chemical etchings. A second 1 μm thick SU-8 layer was deposited and structured to define the electrodes and connecting pads while insulating the metal tracks.

Finally, and differently from the previous article from the authors, where the full fabrication process is described in more detail [18], the metallic sacrificial layer was removed by using an anodic metal dissolution. Now, detachment of the SU-8 structures was performed in a neutral salt solution bath by applying a positive potential to the metallic sacrificial layer. The use of an environmentally friendly media minimizes the delamination of the SU-8 structures, which was a handicap when using a HF-solution to etch a SiO_2_ sacrificial layer. Specifically, aluminum was dissolved by applying −0.2 V against an Ag/AgCl reference electrode. Under this potential the aluminum layer is dissolved in a very short time (less than 10 min), while the chromium it is not dissolved. Therefore, a proper electrical contact is ensured along the whole wafer.

Individual SU-8 sensor devices were connected to a printed circuit board (PCB) using zero insertion force (ZIF) connectors. In Figure 2, an impedance sensor ready for being used is shown. As it can be observed, the PCB was waterproof-protected with an epoxy resin (Epo-Tek OG147-7) in order to facilitate the sensor manipulation. Moreover, the electrodes were electrochemically coated with a porous layer of platinum black [19] in order to increase its specific surface and, therefore, decrease the electrode impedance value. In this work, all the experiments have been performed using the 5 mm electrode configuration (*i.e.*, total sensor width, W_s_ = 5 mm), which was demonstrated to be the most suitable configuration to assess the epithelial permeability [18].

### 2.2. Sensor Characterization

The electrode–electrolyte impedance has been measured in order to evaluate the effect of the electrode modification process. For that, the impedance sensor was immersed in a physiological saline solution (0.9%wt. NaCl, resistivity at 298 K = 0.7 Ωm) where the electrode–electrolyte impedance was measured versus a platinum reference electrode (Radiometer Analytical, Villeurbanne, France) in the 100 Hz to 100 kHz frequency range. Measurements were performed using a custom-made bipolar impedance analysis system [6,20], which allows the measurement of all electrodes simultaneously.

To evaluate the parasitic effects of the electrode–electrolyte interface on the measured impedance, tetrapolar impedance measurements were also performed in the same saline solution. A custom-made tetrapolar impedance analysis system [21] was used to determine the frequency band where the parasitic effects of the electrode–electrolyte interface are depreciable (100 Hz to 1 MHz). Moreover, to verify the stability of the electrodes, these measurements were performed before and after each series of *in vivo* experiments.

### 2.3. Experimental Procedures

In this work, assessment of the corneal epithelium permeability has been performed by monitoring the healing process of rabbit corneas after being wounded. Experiments have been done in 50 New Zealand white rabbits which were anaesthetized with a single intramuscular injection of 50 mg/kg of ketamine (Imalgene 1000^®^, Merial, Lyon, France) plus 7 mg/kg of Xilacine (Rompun^®^, Bayer, Leverkusen, Germany). Then, both eyes were kept open by a blepharostat and a circular wound was performed in the central corneal epithelium by applying a 6 mm diameter paper disc which was previously soaked during 30 s with 10 μL of *n*-heptanol. After removing the paper disk, the cornea was rinsed with 10 mL of sterile saline solution. It has been reported that this protocol generates an epithelial wound with little or even no damage to the underlying stroma [22].

To evaluate the wound healing, the impedance measurements performed with the proposed sensor have been compared with corneal fluorescein staining images. In brief, 40 μL of fluorescein (0.1%) solution was applied to the eye, which was followed by photography under a cobalt filter-attached slit-lamp [23]. The obtained images were analyzed with image analysis software (ImageJ) to determine the area affected by the wound. From this analysis it can be obtained the ratio between the area affected by the wound and the total corneal area. This value, FITC, quantitatively expresses the information obtained from the fluorescein staining images.

For the impedance measurements, the above-described home-made tetrapolar impedance analysis system together with a home-made multiplexer was used. With this system it was possible to acquire the impedance measurements of the four different electrode configurations in an automatic fashion.

Both measurements were carried out on the same eye. Firstly, the impedance was evaluated using the flexible sensor and afterwards, the cornea was fluorescently stained. This procedure was repeated before wounding, to obtain the basal values, and after wounding (15 min and 6, 24 and 48 h).

## 3. Results and Discussion

**Figure 3.**
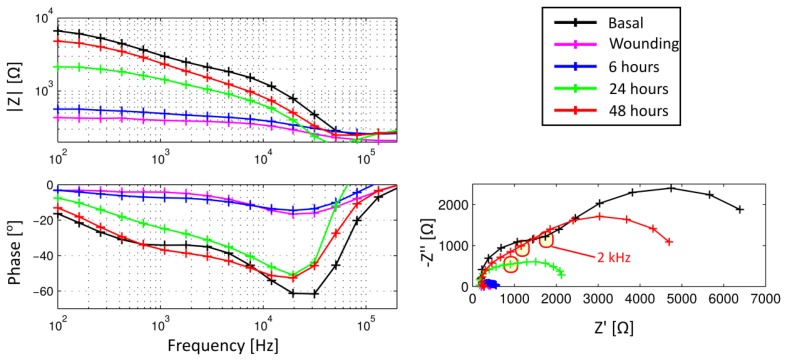
Experimental impedance measurements for the eyes, before and after being wounded. (left) Bode and (right) Nyquist representation of the mean values for each group.

The results presented in this work have been performed in collaboration with the pharmaceutics company SALVAT S.A. Impedance measurements performed during corneal epithelium wound healing process are shown in Figure 3 in both Bode and Nyquist representation. These results are presented by plotting the mean of all the measurements for each group performed with the 5 mm electrode configuration. As expected, an increase, in both the impedance module and phase, related to the elapsed time from wounding can be clearly observed. Moreover, the basal state is not even reached after 48 h.

**Figure 4.**
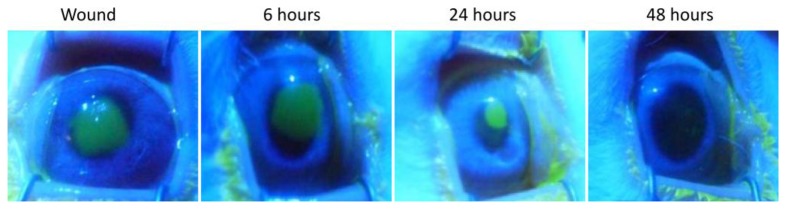
Fluorescent staining images, wherein the corneal epithelium wound appears in bright green upon illumination by blue cobalt light. It can be observed the evolution of the healing process along the time.

Figure 4 shows the images of the fluorescein staining during the evolution of the wound healing process. There, it is interesting to note that the cornea appears to be healthy after 48 h of wounding. Moreover, and taking into account that the fluorescein evaluation was performed after the impedance measurement, any structural alteration produced by the sensor application would be observed in the fluorescein staining images. Therefore, it can be stated that the impedance sensor does not damage the corneal surface.

**Figure 5.**
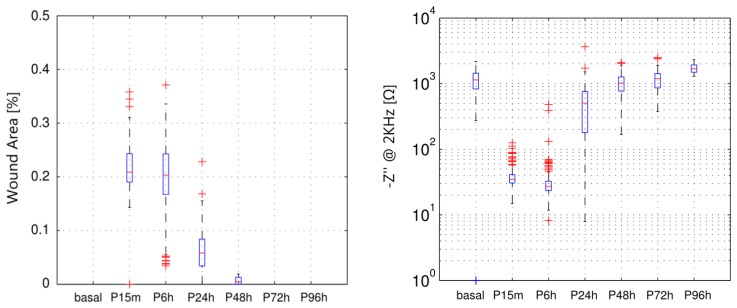
Boxplot representations of the measured wound affected area (FITC) (**left**) and the value of the imaginary part of the impedance measure at 2 kHz (**right**). Measurements have been performed on the same eye, before wounding (basal) and after 15 min, 6 h, 24 h, 48 h, 72 h and 96 h.

To quantitatively expresses the information obtained from the fluorescein staining images, the ratio between the area affected by the wound and the total corneal area (FITC) has been measured. In Figure 5, the impedance values from measurements performed before and after the wounding are shown and compared to the FITC values obtained at the same time. In particular, values of imaginary part measured at 2 KHz, which was proposed as an indicator of the epithelial permeability [18], are shown in a boxplot representation for each measured time. It is interesting to note that the value of the proposed indicator after wounding is almost null. This fact denotes that the proposed indicator is mainly related to the corneal epithelium barrier function since the *n*-heptanol removes all the epithelial cells and, therefore, no barrier exists. It can be also observed that the value of the indicator after 48 h is lower than the basal measurement. On the contrary, in both fluorescein tests performed after 48 h (optical image shown in Figure 4 and FITC measurement in Figure 5, left) the corneal epithelium appears to be healthy. Therefore, it can be stated that although fluorescein test has more accuracy to evaluate the area affected by the wounding, its accuracy is substantially reduced to evaluate the epithelial permeability in advanced stages of the healing process. In those stages, our proposed method is more sensitive to assess the epithelial permeability; then, it could be used as a good complement to the more subjective *in vivo* measurements of the fluorescein test.

Moreover, these results can be compared with the results reported by Fukuda and Sasaki, who used a non-invasive method in a similar experimental procedure [15]. Fukuda and Sasaki performed the impedance measurements at 1 kHz with two circular gold wire electrodes placed on the corneal surface. Their results, which were presented as the percentage variation of the impedance modulus, showed a temporal evolution similar to the results presented here. However, the variation measured after wounding is much lower than the variation obtained with the method presented in this work (50% variation reported by Fukuda and Sasaki *vs.* 96,7% variation obtained in our model). Since the range of the variability is similar in both methods, the higher variation obtained with our method indicates that it has a higher sensitivity than the one presented by Fukuda and Sasaki.

## 4. Conclusions

In previous studies, the feasibility of a new model to non-invasively assess the corneal epithelial permeability through tetrapolar impedance measurements was demonstrated by using a flexible sensing device. Here, a variation on the fabrication process of the SU-8 based sensing device is reported in order to improve the fabrication yield. Moreover, the usability of the flexible sensor has been demonstrated by evaluating the corneal permeability during the healing process of wounded corneas. The impedance results obtained with the fabricated sensing device have been compared with their corresponding fluorescein staining images, which is the most extensively used clinical method to evaluate the corneal permeability. The results show that 48 h after being wounded, corneas which appear to be healthy when being fluorescein stained, have not recovered its basal state if the impedance measurements are taken into account. This demonstrates that the accuracy in evaluating the corneal permeability of our proposed method is higher. Then, this method shows a great value for being used in advanced stages of the healing process. These results, together with the device usability, places the presented method as a reliable complement for the more subjective *in vivo* evaluation performed with fluorescein staining.
